# Immune cell signature in non-ischemic heart failure indicates chronic systemic immune activation with features of immunosenescence

**DOI:** 10.1186/s12979-026-00568-6

**Published:** 2026-03-31

**Authors:** Lukas Baumhove, Gwenny M.P.J. Verstappen, Frederik E. Deiman, Just Dronkers, Wayel H. Abdulahad, Kornelis van der Geest, Elisabeth Brouwer, Adriaan A. Voors, Peter van der Meer, Nils Bomer

**Affiliations:** 1 Department of Cardiology, University of Groningen, University Medical Center Groningen, Groningen, The Netherlands; 2 Department of Rheumatology and Clinical Immunology, University of Groningen, University Medical Center Groningen, Groningen, Netherlands; 3 Department of Clinical Sciences, College of Medicine, University of Sharjah, Sharjah, UAE; 4 Department of Pathology and Medical Biology, University of Groningen, University Medical Center Groningen, Groningen, Netherlands

**Keywords:** Immunosenescence, Heart failure, Flow cytometry, Monocytes, T-cells, Dendritic cells

## Abstract

**Aims:**

Heart failure (HF) is characterized by systemic inflammation and adverse myocardial remodeling involving immune cell activation. Nonetheless, effective immunomodulatory therapies remain lacking. We aimed to characterize the composition and effector function of immune cells in patients with non-ischemic HF.

**Methods and results:**

Peripheral blood mononuclear cells were isolated from patients with non-ischemic HF (*n* = 19) and controls without HF (*n* = 19). Using multiparametric flow cytometry, immune cell populations—including monocyte subsets, dendritic cells (DCs), and T-cell subsets—were characterized alongside expression of pattern recognition receptors and immune checkpoint molecules. Monocyte effector functions were assessed via intracellular cytokine staining after lipopolysaccharide (LPS) stimulation. In HF patients, circulating monocytes were increased, whereas the proportion of non-classical (CD14⁻CD16⁺) monocytes was reduced. Monocytes showed higher PD-L1 expression, and intermediate/non-classical subsets had increased TLR4. Following LPS stimulation, monocyte cytokine production (IL-1β, IL-6, TNF-α) was unchanged. Lower frequencies of non-classical monocytes correlated with reduced left ventricular ejection fraction but not with NT-proBNP. Circulating DCs were elevated. T-cell analysis revealed increased CD4⁺ effector Th and IL-17-producing (Th17) cells and fewer regulatory T-cells. To explore whether systemic immune alterations are reflected in cardiac infiltration, myocardial tissue from a subset of HF patients (*n* = 7) and non-failing controls (*n* = 4) was analyzed by immunohistochemistry. Despite the systemic immune alterations, large myocardial immune cell infiltrates were not readily apparent in end-stage HF tissue.

**Conclusions:**

Non-ischemic end-stage HF is associated with systemic immune alterations indicative of chronic activation with features suggestive of immunosenescence. Without overt corresponding myocardial immune cell infiltrates, these findings emphasize the relevance of systemic immune alterations in non-ischemic end-stage HF.

**Graphical Abstract:**

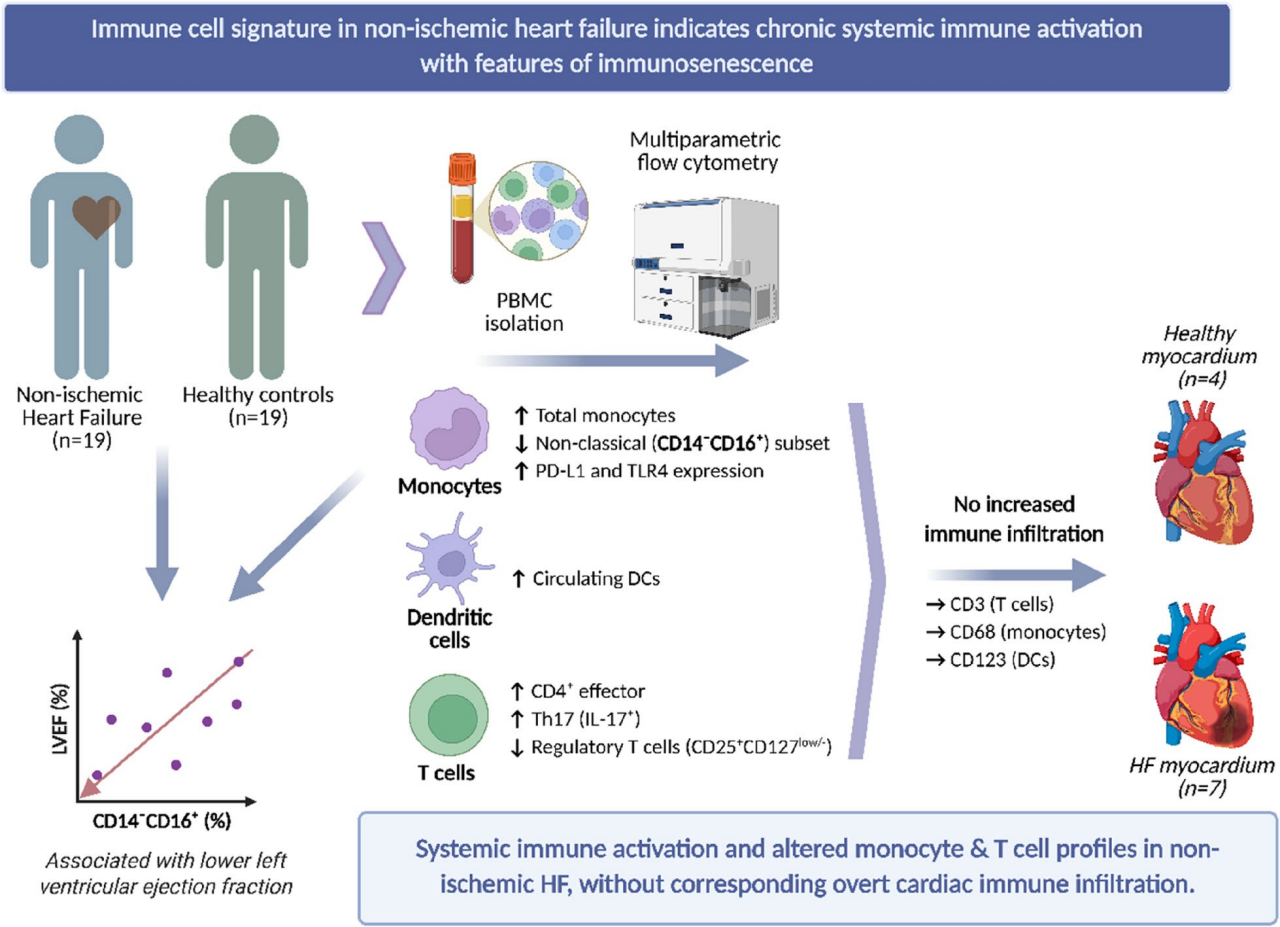

**Supplementary Information:**

The online version contains supplementary material available at 10.1186/s12979-026-00568-6.

## Introduction

Inflammation is a critical factor in the pathophysiology of heart failure (HF), as evidenced by elevated circulating levels of inflammatory cytokines and a significant involvement of immune cells in cardiac remodeling [1, 2]. While there is substantial evidence supporting the involvement of inflammation in HF’s pathophysiology, a large gap remains in translating this knowledge into effective immunomodulatory therapies. Current treatments largely focus on hemodynamic, neurohormonal and metabolic pathways, while the intricate role of immune components represents an underused therapeutic domain [3]. A number of studies have indicated that immune cell subsets, including monocytes, dendritic cells and T-cells, may play a role in the inflammatory pathophysiology of heart failure, particularly in the case of ischemic etiology [2, 4, 5]. Monocytes play a pivotal role in cardiovascular health and disease, contributing to cardiac remodeling, pathogen elimination, and tissue repair. However, their excessive inflammatory responses can lead to cardiac fibrosis and heart failure [6]. They represent a crucial part of the innate immune system and differentiate into macrophages, driving detrimental inflammatory processes in myocardial remodeling. However, they are also required for inflammation resolution and tissue repair after cardiac injury [7–9]. One study showed that patients with acute heart failure exhibited elevated levels of intermediate and non-classical monocytes [10]. Another crucial cellular compartment of the innate immune system are dendritic cells (DC). DCs direct innate and adaptive immune processes and encompass diverse subpopulations that influence acute and chronic inflammatory responses and also contribute to involved immune processes of cardiovascular diseases such as the regulation of monocyte and macrophage homeostasis [11, 12]. DCs are increased in blood of patients with chronic HF and they act as activators and recruiters of T-cells in murine models of heart failure [13–15]. T-cells, as part of the adaptive immune system, also contribute to the response and progression of heart failure by modulating myocardial function [2]. In mice with chronic heart failure, T-cells are globally expanded and several T-cell subtypes influence adverse cardiac remodeling, suggesting an important role of T-cells in heart failure progression [16, 17].

Collectively, the available evidence from animal models and in vitro studies points towards involvement of the immune system in HF pathophysiology. However, comprehensive data on the immune cell composition and cell characteristics in patients with HF, particularly non-ischemic HF, are still lacking. Therefore, we sought to gain a more detailed understanding of the immune signature in peripheral blood and heart tissue of patients with non-ischemic HF.

## Materials and methods

### Study population

Peripheral blood mononuclear cells (PBMCs) were obtained from patients with non-ischemic heart failure due to dilated cardiomyopathy (DCM, *n* = 19) and controls without heart failure (*n* = 19). Controls were selected from a cohort of elderly individuals without HF, which has previously been described [18]. Neither patients nor controls received immunosuppressive treatment or had an ongoing infection. Cytomegalovirus (CMV) status was investigated in all participants. In addition, cardiac tissue samples were obtained from a subset of the included non-ischemic HF patients during left ventricular assist device (LVAD) implantation. These samples were compared with healthy tissue from four donor hearts that could not be transplanted for logistic reasons. The current study is conducted according to the principles of the Declaration of Helsinki (7th revision, October 2013, Fortaleza, Brazil) and in accordance with the Dutch Medical Research Involving Human Subjects Act (*Wet medisch-wetenschappelijk onderzoek met mensen*). The scientific advisory board of the University Medical Centre Groningen provided ethical approval for the collection of human heart tissues (protocol numbers 2020.326 and 2020.327, UMCG Research Register number 202000351; ABR number NL73976.042.20). Written informed consent was obtained. Regarding the control cohort, the study was approved by the institutional review board of the UMCG (METc2012/375) and the Central Committee on Research Involving Human Subjects in the Netherlands (CCMO; ISRCTN64117538).

### Flow cytometry analysis

Flow cytometry was employed to investigate the phenotypes of various immune cell populations, including subsets of monocytes, subsets of dendritic cells, and several subsets of T-cells. In addition, the surface expression of several chemokine receptors, pattern recognition receptors and immune checkpoint molecules was evaluated (supplement table S1). Effector function of monocytes was assessed in a subset of participants (*n* = 10 patients and controls). Flow cytometry experiments were conducted using cryopreserved PBMCs. PBMCs were thawed in Roswell Park Memorial Institute 1640 medium (RPMI, Lonza, Switzerland) + 10% fetal calf serum (FCS). After thawing, cells were divided over five flow cytometry experiments: (1) phenotyping of monocytes and DCs, (2) phenotyping of senescence markers on monocytes, (3) phenotyping of T-cell subsets by surface receptor expression, (4) phenotyping of T-cell subsets by surface markers and intracellular cytokine expression, and (5) analysis of cytokine expression by monocytes. Antibodies included in each experiment are listed in Supplementary Table S1. Cell viability was assessed using propidium iodide (experiment 1) or a fixable viability dye (other experiments). For phenotyping of T-cell subsets by intracellular cytokine expression (experiment 4), PBMCs were stimulated with phorbol 12-myristate 13-acetate (PMA, 50 ng/ml), calcium ionophore A23187 (1.6 µg/ml) in the presence of brefeldin A (10 µg/ml) for 4 h at 37 °C with 5% CO_2_. After washing and staining for surface markers, cells were fixed using FIX & PERM Cell Fixation & Cell permeabilization Kit reagents (Life Technologies, USA), washed, and stained with antibodies for intracellular cytokines. For detection of cytokine expression by monocytes (experiment 5), PBMCs were stimulated with lipopolysaccharides (LPS; Escherichia coli type O26:B6, Sigma, 0.1 µg/ml) for 3.5 h at 37 °C with 5% CO2 in the presence of GolgiStop (BD Biosciences). After washing and staining for surface markers, cells were fixed using Cytofix/Cytoperm Plus reagents (BD Biosciences, USA). Before staining, Fc receptor blocking with TruStain FcX™ (Biolegend, USA) was performed in the monocyte assay. For incubation steps with multiple antibodies, Brilliant Stain Buffer (BD Biosciences) was used. Samples were measured using the Cytek Aurora™ spectral flow cytometer or BD FACSymphony™ flow cytometer. To ensure consistency across different sessions, cytometer setup and tracking beads were employed for normalization. Gates were set based on FMO controls and biological (unstimulated) controls. Examples of the gating strategy for the main results (experiments 1–5) can be found in Supplementary Figure S1-S5.

### Heart tissue immunostaining

Formalin-fixed, paraffin-embedded (FFPE) left ventricular (LV) tissue samples were obtained from seven patients, included in the study population (*N* = 19), with non-schemic, end-stage heart failure (HF) who underwent LVAD implantation. All seven patients presented with a dilated phenotype and severely reduced LVEF, necessitating the implantation of a LVAD. As controls, LV tissue from four non-failing donor hearts was used. Tissue Sect.  (4 μm) were deparaffinized in xylene and rehydrated through graded ethanol series.

Immunohistochemistry for CD3, CD68, and CD123 (plasmacytoid DCs) was performed by the Department of Pathology, University Medical Center Groningen, using standardized protocols as part of their routine diagnostic workflow. In short, FFPE sections were deparaffinized, underwent antigen retrieval, and were incubated with primary antibodies, followed by detection with HRP-conjugated secondary antibodies and DAB chromogen. Counterstaining with hematoxylin was performed, and stained sections were automatically scanned using a Nanozoomer 2.0-HT digital slide scanner (Hamamatsu, Japan). Immunofluorescence was performed for CD3 and CD11c on FFPE section. In short, FFPE sections were deparaffinized, underwent antigen retrieval in 10mM TRIS with 1mM EDTA at pH 9 then samples were permeabilized with 0.2% IGEPAL in PBS. Samples were then incubated with anti-CD3 mAb (1:50 in PBS/1%BSA; Mouse; #M7254, Dako) or anti-CD11c mAb (1:50 in PBS/1%BSA; Rabbit; MA5-14520, Thermo Fisher Scientific) for one hour followed by Alexa 555-Red labelled anti-Mouse secondary antibody (1:100 in PBS/1%BSA; Donkey; Invitrogen A11008) or Alexa 555-Red labelled anti-Rabbit secondary antibody (1:100 in PBS/1%BSA; Donkey; ThermoFisher, #A31572), respectively, and WGA-FTTC (1:100 in PBS/1%BSA; Sigma, L4895-2 mg) for 30 min. Samples were mounted in Vectashield Antifade mounting medium with DAPI (Vector laboratories, H-1200-10).

### Statistical analysis

The data were analyzed using GraphPad Prism 9.1.0 and Stata 18.0. Outliers were identified using the Rout method. The Mann–Whitney U test or unpaired t-test was employed to compare two groups when appropriate. Furthermore, Spearman rank correlations were performed. All reported *p*-values are two-sided, and a two-sided *p*-value of less than 0.05 was considered statistically significant.

## Results

**Fig. 1:**
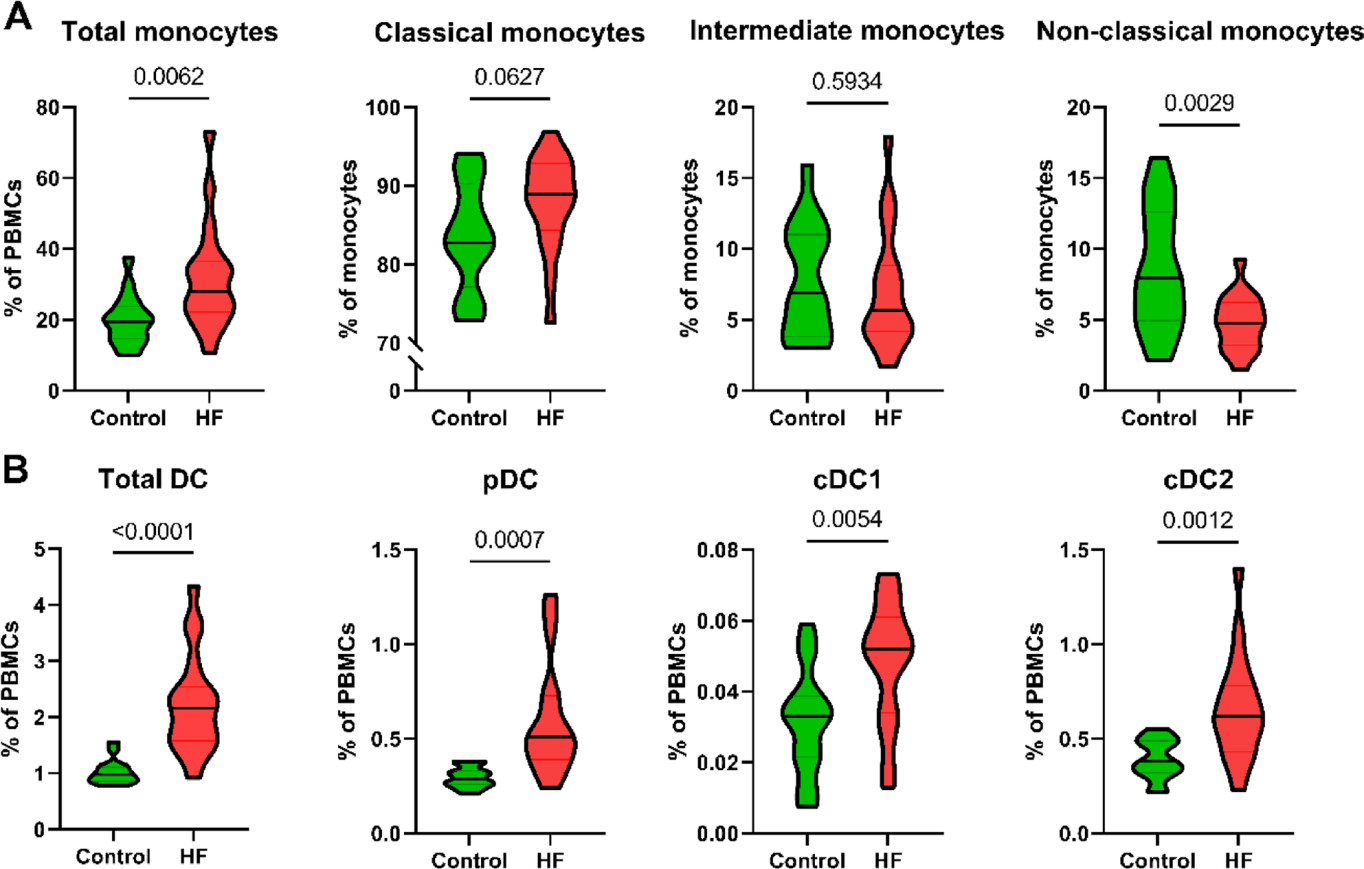
Frequencies of monocytes and dendritic cell subsets in patients with heart failure (HF) and controls. **A** Frequencies of classical monocytes, intermediate monocytes and non-classical monocytes among total monocytes. **B** Frequencies of cDC1, cDC2 and pDCs among total PBMCs. pDC, plasmacytoid dendritic cell cDC1, conventional dendritic cell subset 1; cDC2, conventional dendritic cell subset 2. Violin plots show the distribution (shape), with the median (line) and the interquartile range (grey lines), of biological replicates. *P*-values for differences (shown as numbers in the plots) below 0.05 were considered significant

Table 1 displays the baseline characteristics of the patients with HF (including the 7 patients of which LV tissue was obtained) and controls. In patients with HF, a relative increase in the total monocyte population was observed. Within total monocytes, the non-classical (CD14^−^CD16^+^) subset was decreased compared to controls without HF. Frequencies of classical (CD14^+^CD16^−^) and intermediate (CD14^+^CD16^+^) monocytes did not show significant differences (Fig. 1A). Furthermore, the population of dendritic cells (DCs) was elevated in patients with HF. This increase was consistently observed across plasmacytoid dendritic cells (pDCs), conventional dendritic cell type 1 (cDC1) and conventional dendritic cell type 2 (cDC2), all of which were substantially higher in patients with HF (Fig. 1B).

**Table 1:** Baseline characteristics of patients with HF and controls. BMI, body mass index; CMV, cytomegalovirus; CRP, C-reactive protein; NT-proBNP, N-terminal pro-B-type natriuretic peptide; LVEF, left ventricular ejection fraction; NYHA, New York Heart Association

CHARACTERISTICS	CONTROLS	HEART FAILURE
N	19	19
Age, mean (SD)	67.8 (5.6)	59.5 (11.9)
Female sex	11 (58%)	9 (47%)
BMI, median (IQR)	26.3 (23.9, 27.7)	26.5 (23.5, 28.4)
Smoking	2 (11%)	2 (11%)
Diabetes	3 (16%)	1 (5%)
Hypertension	3 (16%)	3 (16%)
Myocardial infarction	1 (5%)	0 (0%)
Renal disease	0 (0%)	2 (11%)
CMV positive	11 (58%)	9 (47%)
CRP (mg/L), median (IQR)	< 0.5	2.4 (0.9, 3.7)
NT-proBNP (ng/L), median (IQR)	-	2383 (1073, 4765)
LVEF (%), median (IQR)	-	25 (17, 33)
NYHA class		
II	-	10 (53%)
III		6 (32%)
IV		3 (16%)

**Fig. 2:**
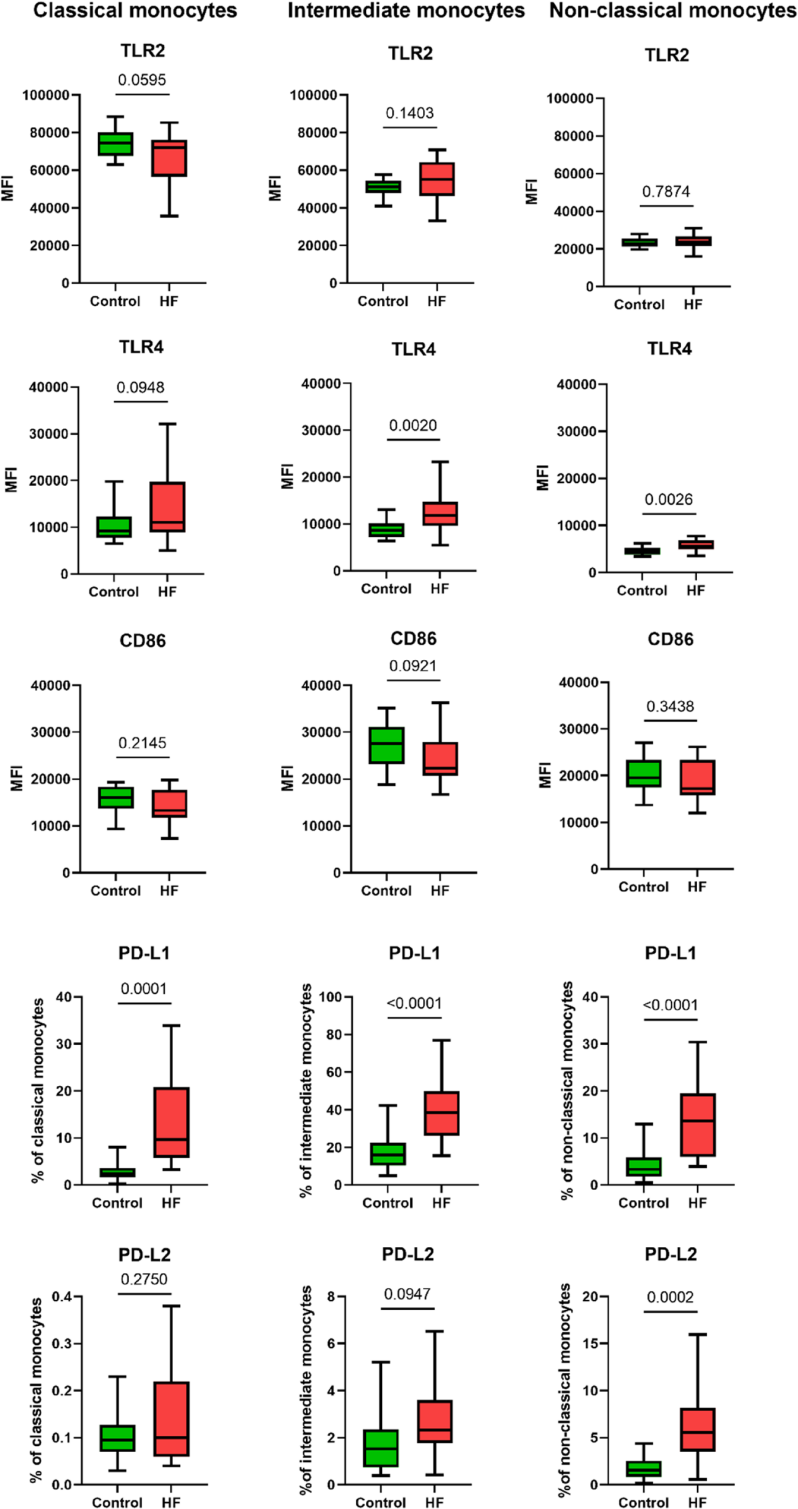
Expression of TLR2, TLR4, CD86, PD-L1 and PD-L2 on monocyte subsets, comparing patients with heart failure (HF) and controls. Boxplots show the median (line), the interquartile range (box), and the minimum and maximum values (whiskers), of biological replicates. *P*-values for differences (shown as numbers in the plots) below 0.05 were considered significant. TLR, toll-like receptor; PD-L1, programmed death-ligand; PD-L2, programmed death-ligand 2

**Fig. 3:**
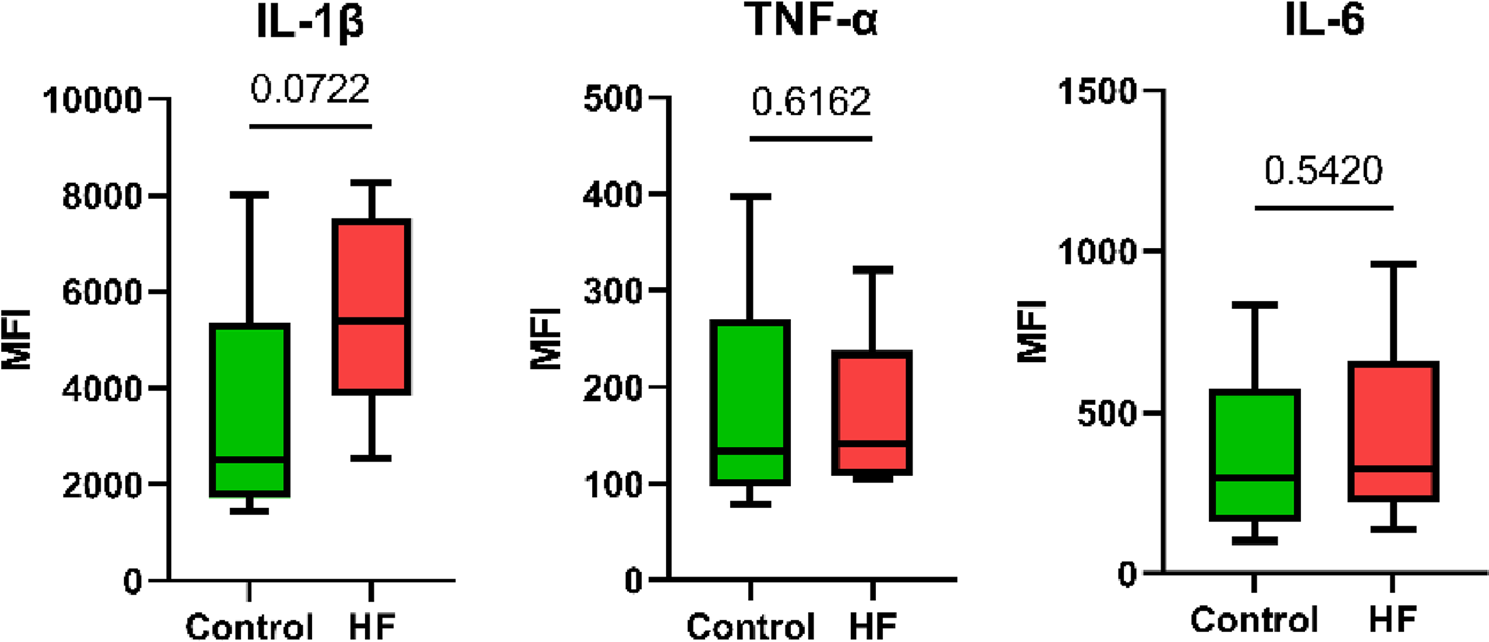
Effector function of monocytes after LPS stimulation, comparing patients with heart failure (HF) and controls. Boxplots show the median (line), the interquartile range (box), and the minimum and maximum values (whiskers) of biological replicates. *P*-values for differences (shown as numbers in the plots) below 0.05 were considered significant. IL-1β, interleukin-1 beta; IL-6, interleukin-6; TNF-α, tumor necrosis factor alpha

To assess the activation state of monocytes and DCs, expression levels of several pattern recognition receptors and immune checkpoint molecules were measured. The expression level of Toll-like receptor 4 (TLR4) was higher on intermediate and non-classical monocytes in patients with HF, while expression levels of Toll-like receptor 2 (TLR2) and CD86 were similar between HF patients and controls across all monocyte subtypes (Fig. 2). Histograms showing median fluorescent intensity values for TLR2, TLR4, and CD86 in different monocyte subsets are shown in Supplementary Figure S6. All subsets of monocytes exhibited increased programmed death-ligand 1 (PD-L1) expression, while non-classical monocytes additionally showed increased programmed death-ligand 2 (PD-L2) expression. Following LPS stimulation of monocytes, intracellular interleukin-1 beta (IL-1β), IL-6, and tumor necrosis factor alpha (TNF-α) levels were similar between groups (Fig. 3).

**Fig. 4:**
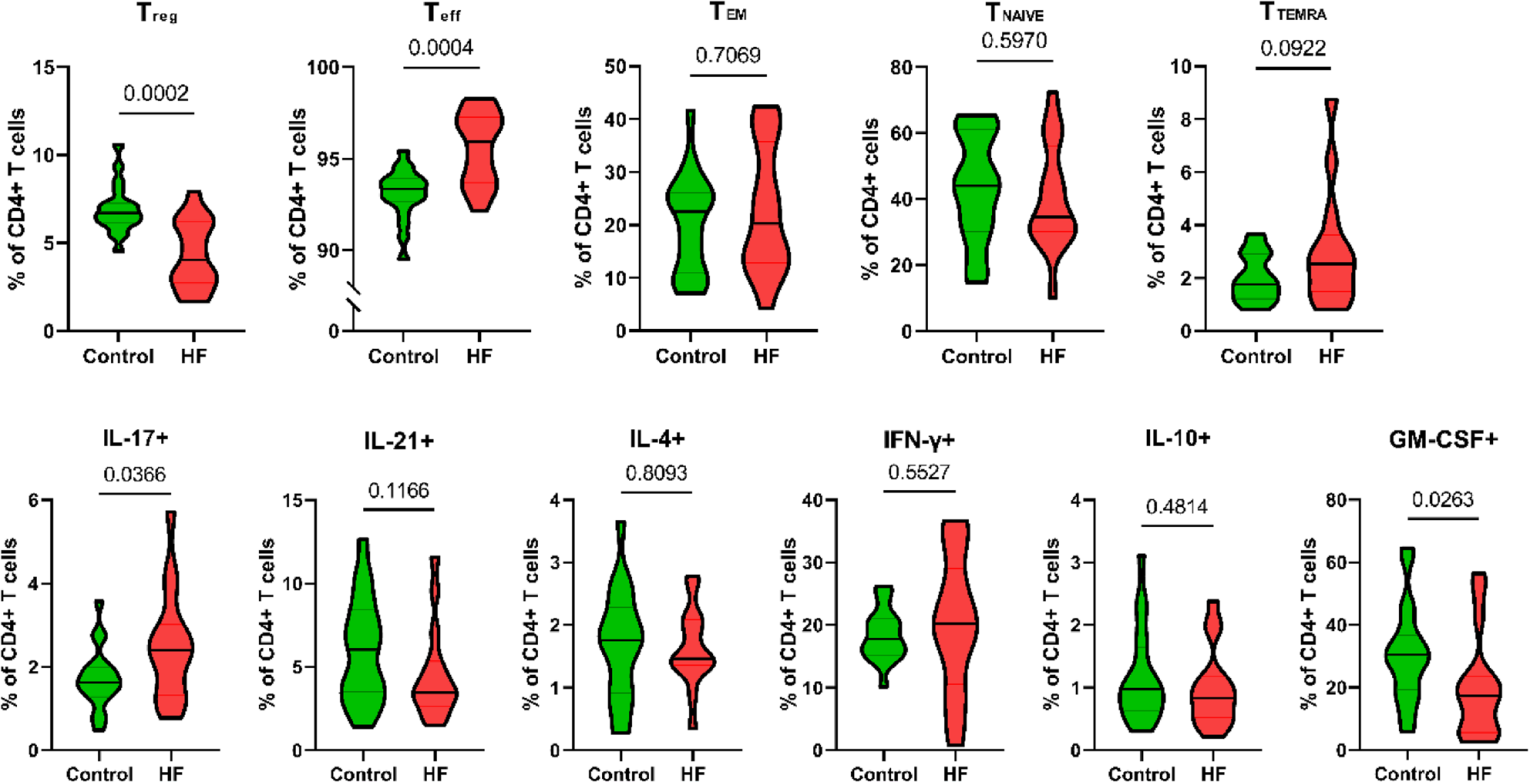
Frequencies of CD4 + T-cell subsets in patients with heart failure (HF) and controls, comparing patients with heart failure (HF) and controls. Violin plots show the distribution (shape), with the median (line) and the interquartile range (grey lines) of biological replicates. *P*-values for differences (shown as numbers in the plots) below 0.05 were considered significant. Treg, T regulatory cells; Teff, T effector cells; Tem, T effector memory cells; Tnaive, T naive cells; Temra, T effector memory cells

Analyses of T-cells revealed an increase in CD4 + effector T-cells (CD45RO^+^CCR7^−^) and a decrease in regulatory CD4 + T-cells (Tregs; CD25^+^CD127^low/−)^) in patients with HF compared to controls. Additionally, IL-17-producing CD4 + T-cells were elevated, while granulocyte-macrophage colony-stimulating factor (GM-CSF) producing CD4 + T-cells were reduced. No significant differences were observed in the frequencies of CD4 + naive (Tnaive), central memory (Tcm), and terminal effector memory (Temra) CD4 + T-cells. Similarly, IL-21-, IL-4, interferon-gamma (IFN-γ)-, and IL-10-producing CD4 + T-cells showed no significant differences (Fig. 4).

Spearman correlation analyses revealed a negative correlation between classical monocytes and LVEF (left ventricular ejection fraction) and a positive correlation between non-classical monocytes and LVEF (Table 2). Thus, patients with decreased frequencies of non-classical monocytes had lower LVEF.

**Table 2:** Spearman correlations with left ventricular ejection fraction (LVEF) and N-terminal pro b-type Natriuretic Peptide (NT-proBNP) in patients with HF. *P*-values for correlations below 0.05 were considered significant

Cell type	*r* with LVEF	*p*-value	*r* with NT-proBNP	*p*-value
Classical monocytes	-0,49	0,0353	0,21	0,3789
Intermediate monocytes	0,39	0,0943	-0,18	0,4636
Non-classical monocytes	0,64	0,0029	-0,28	0,2383
Dendritic cells	0,17	0,4966	0,03	0,8922

**Fig. 5:**
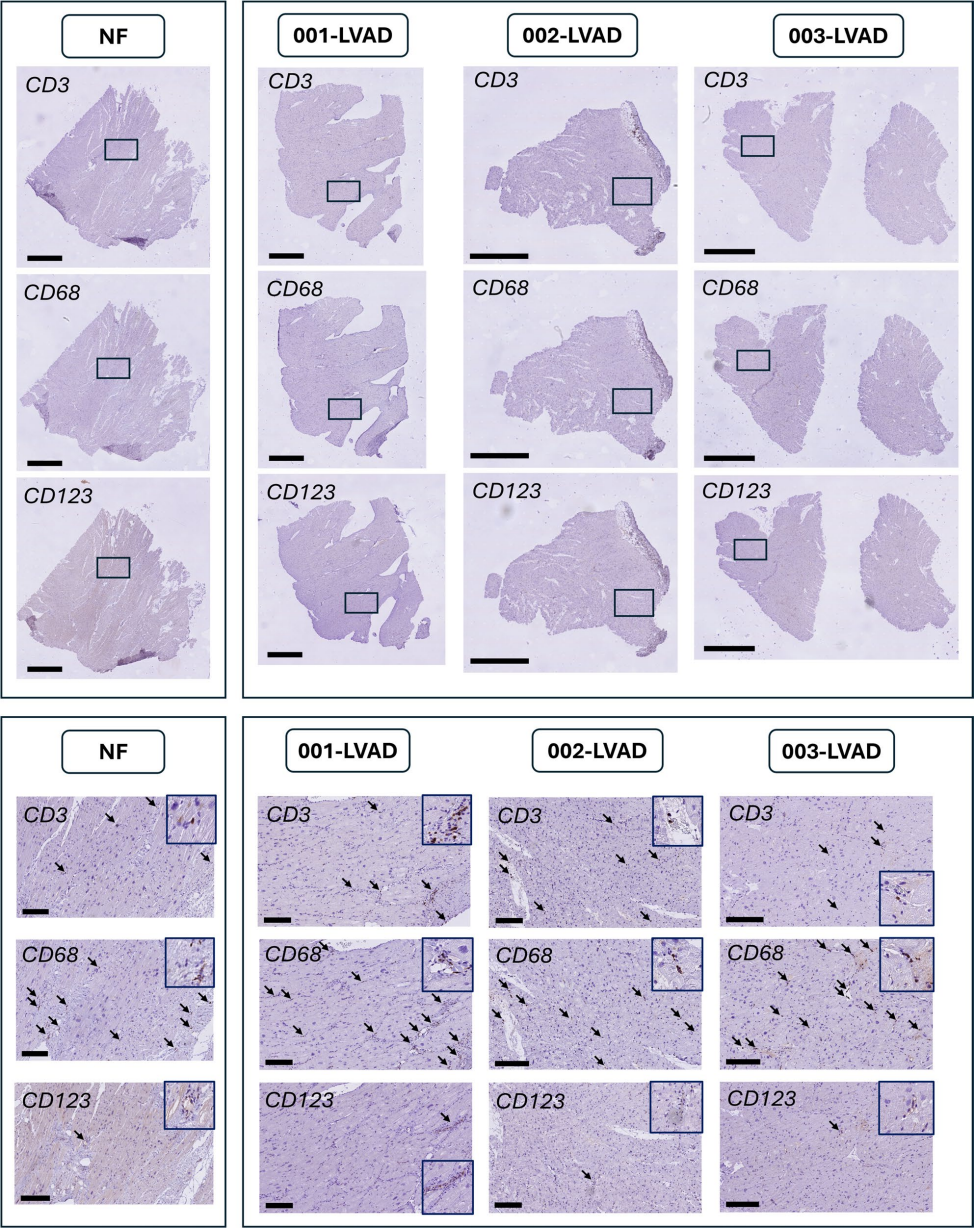
Representative histology images of immune cell staining in non-failing control (NF) and in patients with end-stage heart failure (LVAD) show no altered presence of either T-cells (CD3), macrophages (CD68), or plasmacytoid dendritic cells (CD123) in end-stage HF tissue compared to healthy controls (staining were performed in *n* = 4 control and *n* = 7 LVAD samples), scale bar: 2000 mm. Additional 10x zoom-in (sizebars = 200 μm) with arrows indicating examples of positive staining. An additional zoom-in was added to reflect the positive signal at cell level

Next to the peripheral blood samples, we also obtained apical LV tissue samples from seven patients included in this study who underwent LVAD implantation. To assess whether observations in the circulation mirrored tissue specific alterations, we performed immunohistology in heart tissues of this subset of patients. To compare the results non-failing heart, we obtained LV tissue from four donor hearts that were not transplanted due to logistical reasons. Notably, immunohistochemistry staining for selected immune cell markers (CD3, CD68 and CD123) showed no overt immune cell infiltrates for all tissue samples (Fig. 5). Similar neutral results were obtained after performing immunofluorescence targeting similar immune cells ((CD3, CD68 and CD11c)). Accordingly, no clear differences in the presence of either T-cells (CD3), macrophages (CD68), or plasmacytoid dendritic cells (CD123) could be detected between end-stage HF tissue and non-failing control tissues, despite substantial differences observed in the same immune cell populations (or their precursors) in peripheral blood.

## Discussion

This study provides novel insights into the immunological landscape of non-ischemic heart failure (HF), revealing distinct alterations in peripheral immune cell populations. We observed specific changes in monocyte subsets, dendritic cells (DCs), and CD4⁺ T-cell populations, particularly within the Tregs and Th17 compartments. Moreover, monocytes from HF patients exhibited elevated expression of the inhibitory immune checkpoint molecule PD-L1. These findings collectively suggest a shift toward chronic inflammation which may accelerate immune ageing processes. Notably, despite these systemic immune alterations, no significant immune cell infiltration was detected in apical left ventricular (LV) tissue from the same patients, highlighting the complexity of immune processes in HF and the importance of performing spectral flow cytometry to provide deep phenotyping.

In line with previous studies, patients with HF showed a relative increase in total circulating monocytes and a pronounced reduction in non-classical monocytes [6, 19]. The proportion of classical and intermediate monocytes was similar between patients with HF and controls. Non-classical monocytes are typically involved in guarding endothelial integrity and resolving inflammation in damaged tissue [20, 21]. A reduction in their numbers may therefore suggest an impaired capacity to regulate inflammatory responses effectively in HF. As antibacterial defense mechanism, all monocyte subsets express TLR2 and TLR4 as key pattern recognition receptors [22]. While expression levels of TLR2 by monocyte subsets were similar between groups, there was a notable increase in the surface expression of TLR4 in both intermediate and non-classical monocytes of patients with HF compared to controls. TLR4 is a critical mediator of innate immunity and has been implicated in maintaining chronic inflammation in HF and post-myocardial infarction states [19, 23, 24, 25]. Beyond bacteria, endogenous damage-associated molecular patterns can bind to TLR4 and initiate cell activation. Our results suggest that increased TLR4 expression within specific monocyte subsets may contribute to sustained immune activation in HF.

We also found a consistent upregulation of programmed death-ligand 1 (PD-L1) across all monocyte subsets and a subset-specific increase of PD-L2 in non-classical monocytes. The PD-1/PD-L1 pathway exerts a broad range of immunoregulatory functions, is implicated in a number of diseases such as autoimmunity and tumor immunity, and is of particular importance in the suppression of immune response [26]. Moreover, the PD-1/PD-L1 immune checkpoint axis plays a pivotal role in the accumulation of senescent cells and the associated inflammatory processes that occur with aging [27]. The expression of PD-L1 is stimulated by age-related processes, is markedly increased in senescent cells and the activation of the PD-1 receptor on T-cells promotes a state of immunosenescence [28]. These findings indicate an important role of the PD-1/PD-L1 axis in HF. The increased expression of PD-L1 may be indicative of enhanced immune suppression and/or immunosenescence, potentially induced by a chronic inflammatory environment in these patients. Thus, while the present study was not designed to comprehensively characterize immunosenescence, several of the observed immune alterations are consistent with immune ageing-related changes described in previous studies. However, the present study was not specifically designed to comprehensively characterize immunosenescence, and therefore senescence markers were not systematically assessed.

Despite these surface marker alterations, including increased expression of TLR4 by intermediate and non-classical monocytes, stimulation of monocytes with the TLR4 ligand LPS yielded no differences in IL-1β, IL-6, or TNF-α production between HF patients and controls. This suggests that the inflammatory response capacity of monocytes, at least in terms of these key cytokines, is not inherently altered in HF. Nevertheless, previous transcriptomic data have revealed a pro-inflammatory transcriptional shift in HF monocytes [29], indicating that more subtle or non-canonical inflammatory mechanisms may be involved in the inflammatory pathophysiology of HF.

In addition to monocyte alterations, patients with HF displayed elevated frequencies of circulating DCs, including plasmacytoid DCs (pDCs), conventional DC1 (cDC1), and conventional DC2 (cDC2). DCs are essential for initiating and modulating immune responses, and their expansion may reflect persistent immune activation or unresolved tissue injury contributing to chronic inflammation [30, 31]. While cardiac DC depletion has been associated with worse LV function in dilated cardiomyopathy (DCM) [32], other studies show increased DC infiltration in both acute and chronic HF models [33, 34]. Experimental data further underscore the regulatory role for cDC2 in modulating myocardial inflammation [35, 36]. Our findings extend these observations to the peripheral compartment and suggest that circulating DCs may contribute to systemic inflammation in HF.

T-cell subset analysis further revealed a shift toward a pro-inflammatory phenotype. Regulatory T-cells (Tregs), essential for immune homeostasis and inflammation resolution [37, 38], were significantly reduced in HF patients. Tregs interact with monocytes and dendritic cells in HF models and exert a protective function in ischemic HF by reducing inflammation and enhancing macrophage survival [5, 39]. Their depletion suggests an impaired ability to counterbalance inflammation. Concurrently, the increase of Th17 cells in HF patients may further exacerbate this inflammatory state. Th17 and Treg cells share a common requirement for TGF-β, and increased levels of IL-6 may shift the balance towards Th17 cell differentiation at the expense of Tregs [40]. Th17 cells are known for producing IL-17, a cytokine that promotes inflammation and has been implicated in various autoimmune and inflammatory diseases [41–43]. The reduction in Tregs and the increase in Th17 cells suggest a potential shift towards an inflammatory T-cell phenotype in patients with HF. Several IL-17 antagonists are currently being developed for diseases such as psoriasis and secukinumab has been shown to also have beneficial effects on myocardial function [44, 45].

To explore whether peripheral immune dysregulation is reflected by apparent changes at the myocardial tissue level, we analysed paired LV tissue from seven HF patients and compared it to four non-failing controls. Using immuno-histochemistry and -fluorescence to detect T-cells (CD3), macrophages (CD68) and pDCs (CD123/CD11c), no overt differences in immune cell staining were detected in failing myocardium compared to controls, despite pronounced alterations in the peripheral immune signature. These findings suggest that, at the level of detection provided by immunohistochemistry in end-stage tissue, peripheral immune activation in HF does not necessarily translate directly into overt myocardial immune cell infiltration in end-stage HF. Interestingly, recent spatial transcriptomic and immunofluorescence analyses of human HFpEF myocardium similarly reported normal myocardial immune cell abundance without evidence of substantial immune cell expansion, supporting the concept that systemic immune alterations do not necessarily translate into increased immune cell infiltration in myocardial tissue [46].

Together, the results of this study suggest a dysregulated immune state in HF at the systemic cellular level characterized by innate immune activation, checkpoint-mediated immunosuppression and an imbalance within CD4 + T-cell subpopulations. This may open the door to targeted immunomodulatory interventions. TLR4 inhibition represents a potential strategy to mitigate chronic inflammation in HF [47]. Likewise, modulating the PD-1/PD-L1 axis could help restore immune regulation without broadly suppressing host immunity [48]. Therapies aimed at modulating DC function may further support immune homeostasis in HF. Finally, approaches to enhance Treg activity or inhibit Th17/IL-17 signalling could help restore the Treg/Th17 axis and limit inflammation [49]. Collectively, these strategies may offer a more tailored approach to immunomodulation in HF, minimizing the risks of global immune suppression.

### Limitations

This study provides a comprehensive overview of the immune signature of patients with HF compared to a well-matched control group. Albeit the groups are not age-matched, the control group shows lower circulating immune activation, despite being older and having a similar burden in comorbidities other than HF. However, several limitations must be acknowledged. The small sample size limits the statistical power and generalizability of the findings. Furthermore, the study focuses on associations and therefore cannot establish causality. While our choice of immune markers was based on current evidence, it does not encompass the full immune molecule repertoire, and other relevant pathways may have been missed. The use of cryopreserved PBMCs may have introduced variability due to differential cell loss during isolation or the freezing and thawing process. Lastly, tissue-level immune alterations were assessed in a relatively small number of end-stage diseased explanted myocardium and with a small set of markers, limiting the detection of other cell types and low-grade or regionally confined immune infiltration. Furthermore, tissue-level differences may not be readily captured by immunostaining at end-stage HF.

## Conclusion

This study provides characterization of systemic immune alterations in non-ischemic HF, including differences in monocytes, DCs, and T-cell subsets suggestive of chronic inflammation with features of immunosenescence. Despite these pronounced peripheral findings, using immunostaining, myocardial immune cell infiltration was not apparent in end-stage HF tissue. Together, these findings emphasize the complexity of immune processes in HF and highlight the relevance of systemic immune dysregulation in HF pathophysiology. Further research is needed to elucidate how peripheral immune signatures relate to myocardial immune processes across different stages of disease.

## Supplementary Information

Supplementary Material 1. Table S1. Antibodies included in the experiments.

Supplementary Material 2: Figure S1. Gating strategy monocytes and dendritic cell subsets. First, lymphocytes and monocytes were selected based on their cell size (forward scatter; FSC) and internal complexity or granularity (side scatter; SSC). Then, live cells were selected based on negativity for propidium iodide and single cells were selected based on proportional area (FSC-A) and height (FSC-H). To exclude B cells, T cells, and natural killer cells, cells expressing CD3, CD8, CD19, CD56, and/or high levels of CD4 were excluded. Monocytes and dendritic cells (DCs) were then selected based on positivity for HLA-DR. Monocytes were subdivided into classical monocytes (CD14^+^CD16^−^), intermediate monocytes (CD14^+^CD16^+^), and non-classical monocytes (CD14^−^CD16^+^). DCs were defined as Lin^−^HLA-DR^+^CD14^−^CD16^−^ cells and subdivided into the following subsets: CD11c^+^ conventional DCs (CD141^+^ cDC1 and CD1c^+^ cDC2) and CD123^+^ plasmacytoid DCs. Figure S2. Gating strategy monocytes for assessment of PD-L1 and PD-L2 positive cells. (A) First, monocytes were selected based on their cell size (forward scatter; FSC) and internal complexity or granularity (side scatter; SSC). Then, single cells were selected based on proportional width (FSGW) and height (FSGH) and live cells were selected based on negativity for the fixable viability dye. To exclude neutrophils, T cells, and natural killer cells, cells expressing CD15 and CD16, CD3, or CD16 in absence of CD86 were excluded, respectively. Monocytes were subdivided into classical monocytes (CD14^+^CD16^−^), intermediate monocytes (CD14^+^CD16^+^), and non-classical monocytes (CD14^−^CD16^+^). (B) Within these monocyte subsets, positive cell frequencies for PD-L1- and PD-L2 were assessed. The expression pattern of these proteins on total monocytes is shown for one representative control and one patient with heart failure (HF). Figure S3. Gating strategy for T cell subsets based on surface receptor expression. First, lymphocytes were selected based on their cell size (forward scatter; FSGA) and internal complexity or granularity (side scatter; SSGA). Then, live cells were selected based on negativity for the fixable viability dye, followed by gating for CD4^+^CD8^−^ T cells. CD4^+^ T cells were further subdivided into T regulatory cells (CD25^++^CD127^low/−^) and effector cells. Alternatively, CD4^+^ T cells were divided into naïve (CCR7^+^CD45RO^−^), central memory (CCR7^+^CD45RO^+^), effector memory (CCR7^−^CD45RO^+^) and terminally differentiated effector memory (CCR7^−^CD45RO^−^) subsets. The frequency of PD-1^+^ cells was determined for each of these subsets, and a representative example of PD-1 expression on total effector cells is shown in the figure. Figure S4. Gating strategy for T cell subsets based on cytokine expression. (A) First, lymphocytes were selected based on their cell size (forward scatter; FSGA) and internal complexity or granularity (side scatter; SSGA). Then, single cells were selected based on proportional width (FSGW) and height (FSGH) and live T cells were selected based on negativity for the fixable viability dye and positivity for CD3, followed by gating for CD4^+^CD8^−^ T cells. (B) Cytokine expression by stimulated CD4^+^CD8^−^ T cells is shown for one representative control sample. (C) Unstimulated (biological control) samples (from the same donor) were used to set the gates for the stimulated samples. Figure S5.Gating strategy for cytokine expression by LPS-stimulated monocytes. First, lymphocytes and monocytes were selected based on their cell size (forward scatter; FSC) and internal complexity or granularity (side scatter; SSC). Then, single cells were selected based on proportional area (FSC-A) and height (FSC-H) and live cells were selected based on negativity for the fixable viability dye. To exclude B cells, T cells, natural killer cells and neutrophils, cells expressing CD3, CD19, CD56, and/or CD66b were excluded. Monocytes were then selected based on positivity for HLA-DR and positivity for CD16 and/or CD14. Expression patterns of CD14/CD16, IL-1β, IL-6, and TNF-α are shown for a representative unstimulated (blue) and stimulated (red) control sample. Figure S6. Histograms for median fluorescence intensity levels of TLR2, TLR4 and CD86 expression by monocyte subsets. For each surface marker, an overlay of histograms from classical (dark green), intermediate (light green), and non-classical (white) monocytes and T cells (grey; as negative control) from a representative control participant is shown.

## Data Availability

Data supporting this study are available from the corresponding author on reasonable request.

## References

[1] Murphy SP, Kakkar R, McCarthy CP, Januzzi JL. Inflammation in Heart Failure: JACC State-of-the-Art Review. J Am Coll Cardiol. 2020;75:1324–40. 32192660 10.1016/j.jacc.2020.01.014

[2] Adamo L, Rocha-Resende C, Prabhu SD, Mann DL. Reappraising the role of inflammation in heart failure. Nat Reviews Cardiol 2020. 2020;17:5:17:269–85. 10.1038/s41569-019-0315-x 31969688

[3] Markousis-Mavrogenis G, Baumhove L, Al-Mubarak AA, Aboumsallem JP, Bomer N, Voors AA et al. Immunomodulation and immunopharmacology in heart failure. Nat Reviews Cardiol. 2024;21:119–49. 10.1038/s41569-023-00919-6. 10.1038/s41569-023-00919-6 37709934

[4] Farache Trajano L, Smart N. Immunomodulation for optimal cardiac regeneration: insights from comparative analyses. npj Regen Med. 2021;6:8. 10.1038/s41536-021-00118-2. 10.1038/s41536-021-00118-2 PMC7884783 33589632

[5] Prabhu SD, Frangogiannis NG. The Biological Basis for Cardiac Repair After Myocardial Infarction. Circ Res. 2016;119:91–112. 27340270 10.1161/CIRCRESAHA.116.303577 PMC4922528

[6] Shahid F, Lip GYH, Shantsila E. Role of monocytes in heart failure and atrial fibrillation. J Am Heart Assoc. 2018;7(3):e007849. 10.1161/JAHA.117.007849. 10.1161/JAHA.117.007849 PMC5850261 29419389

[7] Heidt T, Courties G, Dutta P, Sager HB, Sebas M, Iwamoto Y, et al. Differential Contribution of Monocytes to Heart Macrophages in Steady-State and After Myocardial Infarction. Circ Res. 2014;115:284–95. 24786973 10.1161/CIRCRESAHA.115.303567 PMC4082439

[8] Bajpai G, Schneider C, Wong N, Bredemeyer A, Hulsmans M, Nahrendorf M, et al. The human heart contains distinct macrophage subsets with divergent origins and functions. Nat Med. 2018;24:1234–45. 29892064 10.1038/s41591-018-0059-x PMC6082687

[9] Nahrendorf M. Myeloid cell contributions to cardiovascular health and disease. Nat Med. 2018;24(6):711–20. 29867229 10.1038/s41591-018-0064-0 PMC7301893

[10] Goonewardena SN, Stein AB, Tsuchida RE, Rattan R, Shah D, Hummel SL. Monocyte Subsets and Inflammatory Cytokines in Acute Decompensated Heart Failure. J Card Fail. 2016;22:358–65. 26705751 10.1016/j.cardfail.2015.12.014 PMC4861694

[11] Christ A, Temmerman L, Legein B, Daemen MJAP, Biessen EAL. Dendritic cells in cardiovascular diseases epiphenomenon, contributor, or therapeutic opportunity. Circulation. 2013;128:2603–13. 24344059 10.1161/CIRCULATIONAHA.113.003364

[12] Dieterlen MT, John K, Reichenspurner H, Mohr FW, Barten MJ. Dendritic Cells and Their Role in Cardiovascular Diseases: A View on Human Studies. J Immunol Res. 2016;2016:5946807. 27088098 10.1155/2016/5946807 PMC4818818

[13] Saleh D, Jones RTL, Schroth SL, Thorp EB, Feinstein MJ. Emerging Roles for Dendritic Cells in Heart Failure. Biomolecules. 2023;13(10):1535. 10.3390/biom13101535. 10.3390/biom13101535 PMC10605025 37892217

[14] Athanassopoulos P, Balk AHMM, Vaessen LMB, Caliskan K, Takkenberg JJM, Weimar W, et al. Blood dendritic cell levels and phenotypic characteristics in relation to etiology of end-stage heart failure: Implications for dilated cardiomyopathy. Int J Cardiol. 2009;131:246–56. 18243370 10.1016/j.ijcard.2007.10.031

[15] Athanassopoulos P, Vaessen LMB, Maat APWM, Balk AHMM, Weimar W, Bogers AJJC. Peripheral blood dendritic cells in human end-stage heart failure and the early post-transplant period: evidence for systemic Th1 immune responses. Eur J Cardiothorac Surg. 2004;25:619–26. 15037281 10.1016/j.ejcts.2004.01.032

[16] Bansal SS, Ismahil MA, Goel M, Zhou G, Rokosh G, Hamid T, et al. Dysfunctional and Proinflammatory Regulatory T-Lymphocytes Are Essential for Adverse Cardiac Remodeling in Ischemic Cardiomyopathy. Circulation. 2019;139:206–21. 30586716 10.1161/CIRCULATIONAHA.118.036065 PMC6322956

[17] Bansal SS, Ismahil MA, Goel M, Patel B, Hamid T, Rokosh G et al. Activated T lymphocytes are essential drivers of pathological remodeling in ischemic heart failure. Circ Heart Fail. 2017;10(3):e003688. 10.1161/CIRCHEARTFAILURE.116.003688. 10.1161/CIRCHEARTFAILURE.116.003688 PMC5331621 28242779

[18] van der Geest KSM, Abdulahad WH, Tete SM, Lorencetti PG, Horst G, Bos NA, et al. Aging disturbs the balance between effector and regulatory CD4 + T cells. Exp Gerontol. 2014;60:190–6. 25449852 10.1016/j.exger.2014.11.005

[19] Wrigley BJ, Lip GYH, Shantsila E. The role of monocytes and inflammation in the pathophysiology of heart failure. Eur J Heart Fail. 2011;13:1161–71. 21952932 10.1093/eurjhf/hfr122

[20] Tahir S, Steffens S. Nonclassical monocytes in cardiovascular physiology and disease. Am J Physiol Cell Physiol. 2021;320:C761–70. 33596150 10.1152/ajpcell.00326.2020

[21] Thomas G, Tacke R, Hedrick CC, Hanna RN. Nonclassical Patrolling Monocyte Function in the Vasculature. Arterioscler Thromb Vasc Biol. 2015;35:1306–16. 25838429 10.1161/ATVBAHA.114.304650 PMC4441550

[22] Oliveira-Nascimento L, Massari P, Wetzler LM. The role of TLR2 ininfection and immunity. Front Immunol. 2012;3:20479. 10.3389/fimmu.2012.00079 PMC3342043 22566960

[23] Lucas K, Maes M. Role of the Toll Like Receptor (TLR) Radical Cycle in Chronic Inflammation: Possible Treatments Targeting the TLR4 Pathway. Mol Neurobiol. 2013;48(1):190–204. 23436141 10.1007/s12035-013-8425-7 PMC7091222

[24] Devaraj S, Dasu MR, Rockwood J, Winter W, Griffen SC, Jialal I. Increased Toll-Like Receptor (TLR) 2 and TLR4 Expression in Monocytes from Patients with Type 1 Diabetes: Further Evidence of a Proinflammatory State. J Clin Endocrinol Metab. 2008;93:578–83. 18029454 10.1210/jc.2007-2185 PMC2243229

[25] Satoh M, Shimoda Y, Maesawa C, Akatsu T, Ishikawa Y, Minami Y, et al. Activated toll-like receptor 4 in monocytes is associated with heart failure after acute myocardial infarction. Int J Cardiol. 2006;109:226–34. 16051384 10.1016/j.ijcard.2005.06.023

[26] Dai S, Jia R, Zhang X, Fang Q, Huang L. The PD-1/PD-Ls pathway and autoimmune diseases. Cell Immunol. 2014;290:72–9. 24908630 10.1016/j.cellimm.2014.05.006

[27] Wang TW, Johmura Y, Suzuki N, Omori S, Migita T, Yamaguchi K, et al. Blocking PD-L1–PD-1 improves senescence surveillance and ageing phenotypes. Nature. 2022;611(7935):358–64. 36323784 10.1038/s41586-022-05388-4

[28] Salminen A. The role of the immunosuppressive PD-1/PD-L1 checkpoint pathway in the aging process and age-related diseases. J Mol Med. 102(6):733–50 10.1007/s00109-024-02444-6. 10.1007/s00109-024-02444-6 PMC11106179 38600305

[29] Abplanalp WT, John D, Cremer S, Assmus B, Dorsheimer L, Hoffmann J, et al. Single-cell RNA-sequencing reveals profound changes in circulating immune cells in patients with heart failure. Cardiovasc Res. 2021;117:484–94. 32311026 10.1093/cvr/cvaa101

[30] Qian C, Cao X. Dendritic cells in the regulation of immunity and inflammation. Semin Immunol. 2018;35:3–11. 29242034 10.1016/j.smim.2017.12.002

[31] Linton PJ, Thoman ML. Immunosenescence in monocytes, macrophages, and dendritic cells: Lessons learned from the lung and heart. Immunol Lett. 2014;162:290–7. 25251662 10.1016/j.imlet.2014.06.017 PMC4256137

[32] Pistulli R, König S, Drobnik S, Kretzschmar D, Rohm I, Lichtenauer M, et al. Decrease in dendritic cells in endomyocardial biopsies of human dilated cardiomyopathy. Eur J Heart Fail. 2013;15:974–85. 23603088 10.1093/eurjhf/hft054

[33] Patel B, Ismahil MA, Hamid T, Bansal SS, Prabhu SD. Mononuclear Phagocytes Are Dispensable for Cardiac Remodeling in Established Pressure-Overload Heart Failure. PLoS ONE. 2017;12:e0170781. 28125666 10.1371/journal.pone.0170781 PMC5268479

[34] Yan X, Anzai A, Katsumata Y, Matsuhashi T, Ito K, Endo J, et al. Temporal dynamics of cardiac immune cell accumulation following acute myocardial infarction. J Mol Cell Cardiol. 2013;62:24–35. 23644221 10.1016/j.yjmcc.2013.04.023

[35] Eriksson U, Ricci R, Hunziker L, Kurrer MO, Oudit GY, Watts TH, et al. Dendritic cell–induced autoimmune heart failure requires cooperation between adaptive and innate immunity. Nat Med. 2003;9(12):1484–90. 14625544 10.1038/nm960

[36] Borght KV, Der, Scott CL, Martens L, Sichien D, Isterdael G, Van, Nindl V, et al. Myocarditis elicits dendritic cell and monocyte infiltration in the heart and self-antigen presentation by conventional type 2 dendritic cells. Front Immunol. 2018;9:389684. 10.3389/fimmu.2018.02714 PMC6258766 30524444

[37] Tang Q, Bluestone JA. The Foxp3 + regulatory T cell: a jack of all trades, master of regulation. Nat Immunol 2008. 2008;9:3:9:239–44. 10.1038/ni1572 PMC3075612 18285775

[38] Collison LW, Chaturvedi V, Henderson AL, Giacomin PR, Guy C, Bankoti J, et al. IL-35-mediated induction of a potent regulatory T cell population. Nat Immunol. 2010;11(12):1093–101. 20953201 10.1038/ni.1952 PMC3008395

[39] Lu Y, Xia N, Cheng X. Regulatory T Cells in Chronic Heart Failure. Front Immunol. 2021;12:732794. 34630414 10.3389/fimmu.2021.732794 PMC8493934

[40] Kimura A, Kishimoto T. IL-6: Regulator of Treg/Th17 balance. Eur J Immunol. 2010;40:1830–5. 20583029 10.1002/eji.201040391

[41] Hu Y, Shen F, Crellin NK, Ouyang W. The IL-17 pathway as a major therapeutic target in autoimmune diseases. Ann N Y Acad Sci. 2011;1217:60–76. 21155836 10.1111/j.1749-6632.2010.05825.x

[42] Chen J, Liao MY, Gao XL, Zhong Q, Tang TT, Yu X, et al. IL-17A Induces Pro-Inflammatory Cytokines Production in Macrophages via MAPKinases, NF-κB and AP-1. Cell Physiol Biochem. 2013;32:1265–74. 24247374 10.1159/000354525

[43] Jovanovic DV, Battista JA, Di, Martel-Pelletier J, Jolicoeur FC, He Y, Zhang M, et al. IL-17 Stimulates the Production and Expression of Proinflammatory Cytokines, IL-β and TNF-α, by Human Macrophages. J Immunol. 1998;160:3513–21. 9531313

[44] Huangfu L, Li R, Huang Y, Wang S. The IL-17 family in diseases: from bench to bedside. Signal Transduct Target Ther. 2023;8:(1):1–22. 37816755 10.1038/s41392-023-01620-3 PMC10564932

[45] Makavos G, Ikonomidis I, Andreadou I, Varoudi M, Kapniari I, Loukeri E, et al. Effects of Interleukin 17A Inhibition on Myocardial Deformation and Vascular Function in Psoriasis. Can J Cardiol. 2020;36:100–11. 31606265 10.1016/j.cjca.2019.06.021

[46] Ye B, Xu Y, Hirashima F, Lewinter MM, Berlo JH, Van, Zile MR, et al. Normal abundance of immune cells in left ventricular myocardium from patients with heart failure and preserved ejection fraction. Eur J Heart Fail. 2026;00:1–6. 10.1093/ejhf/xuag041 41758504

[47] Zhang Y, Liang X, Bao X, Xiao W, Chen G. Toll-like receptor 4 (TLR4) inhibitors: Current research and prospective. Eur J Med Chem. 2022;235:114291. 35307617 10.1016/j.ejmech.2022.114291

[48] Zhang P, Wang Y, Miao Q, Chen Y. The therapeutic potential of PD-1/PD-L1 pathway on immune-related diseases: Based on the innate and adaptive immune components. Biomed Pharmacother. 2023;167:115569. 37769390 10.1016/j.biopha.2023.115569

[49] Linthout S, Van TschöpeC. The Quest for Antiinflammatory and Immunomodulatory Strategies in Heart Failure. Clin Pharmacol Ther. 2019;106:1198–208. 31544235 10.1002/cpt.1637

