# Supplementary material for: Immune cell signature in non-ischemic heart failure indicates chronic systemic immune activation with features of immunosenescence
Source: Immun Ageing. 2026 Mar 31;23:18. doi: 10.1186/s12979-026-00568-6 (PMC13159214; doi:10.1186/s12979-026-00568-6)
Supplement: Supplementary file 2 — Supplementary Material 2: Figure S1. Gating strategy monocytes and dendritic cell subsets. First, lymphocytes and monocytes were selected based on their cell size (forward scatter; FSC) and internal complexity or granularity (side scatter; SSC). Then, live cells were selected based on negativity for propidium iodide and single cells were selected based on proportional area (FSC-A) and height (FSC-H). To exclude B cells, T cells, and natural killer cells, cells expressing CD3, CD8, CD19, CD56, and/or high levels of CD4 were excluded. Monocytes and dendritic cells (DCs) were then selected based on positivity for HLA-DR. Monocytes were subdivided into classical monocytes (CD14+CD16−), intermediate monocytes (CD14+CD16+), and non-classical monocytes (CD14−CD16+). DCs were defined as Lin−HLA-DR+CD14−CD16− cells and subdivided into the following subsets: CD11c+ conventional DCs (CD141+ cDC1 and CD1c+ cDC2) and CD123+ plasmacytoid DCs. Figure S2. Gating strategy monocytes for assessment of PD-L1 and PD-L2 positive cells. (A) First, monocytes were selected based on their cell size (forward scatter; FSC) and internal complexity or granularity (side scatter; SSC). Then, single cells were selected based on proportional width (FSGW) and height (FSGH) and live cells were selected based on negativity for the fixable viability dye. To exclude neutrophils, T cells, and natural killer cells, cells expressing CD15 and CD16, CD3, or CD16 in absence of CD86 were excluded, respectively. Monocytes were subdivided into classical monocytes (CD14+CD16−), intermediate monocytes (CD14+CD16+), and non-classical monocytes (CD14−CD16+). (B) Within these monocyte subsets, positive cell frequencies for PD-L1- and PD-L2 were assessed. The expression pattern of these proteins on total monocytes is shown for one representative control and one patient with heart failure (HF). Figure S3. Gating strategy for T cell subsets based on surface receptor expression. First, lymphocytes were selected based on t [file 12979_2026_568_MOESM2_ESM.docx]

**Immune cell signature in non-ischemic heart failure indicates chronic systemic immune activation with features of immunosenescence**

Short title: Immune cell signature in heart failure

**Supplementary**

**Table S1:** *Antibodies included in the experiments.*

**Figure S1*:*** *Gating strategy monocytes and dendritic cell subsets.* First, lymphocytes and monocytes were selected based on their cell size (forward scatter; FSC) and internal complexity or granularity (side scatter; SSC). Then, live cells were selected based on negativity for propidium iodide and single cells were selected based on proportional area (FSC-A) and height (FSC-H). To exclude B cells, T cells, and natural killer cells, cells expressing CD3, CD8, CD19, CD56, and/or high levels of CD4 were excluded. Monocytes and dendritic cells (DCs) were then selected based on positivity for HLA-DR. Monocytes were subdivided into classical monocytes (CD14^+^CD16^-^), intermediate monocytes (CD14^+^CD16^+^), and non-classical monocytes (CD14^-^CD16^+^). DCs were defined as Lin^-^HLA-DR^+^CD14^-^CD16^-^ cells and subdivided into the following subsets: CD11c^+^ conventional DCs (CD141^+^ cDC1 and CD1c^+^ cDC2) and CD123^+^ plasmacytoid DCs.

**Figure S2**. *Gating strategy monocytes for assessment of PD-L1 and PD-L2 positive cells.* (A) First, monocytes were selected based on their cell size (forward scatter; FSC) and internal complexity or granularity (side scatter; SSC). Then, single cells were selected based on proportional width (FSGW) and height (FSGH) and live cells were selected based on negativity for the fixable viability dye. To exclude neutrophils, T cells, and natural killer cells, cells expressing CD15 and CD16, CD3, or CD16 in absence of CD86 were excluded, respectively. Monocytes were subdivided into classical monocytes (CD14^+^CD16^-^), intermediate monocytes (CD14^+^CD16^+^), and non-classical monocytes (CD14^-^CD16^+^). (B) Within these monocyte subsets, positive cell frequencies for PD-L1- and PD-L2 were assessed. The expression pattern of these proteins on total monocytes is shown for one representative control and one patient with heart failure (HF).

**Figure S3**. *Gating strategy for T cell subsets based on surface receptor expression.* First, lymphocytes were selected based on their cell size (forward scatter; FSGA) and internal complexity or granularity (side scatter; SSGA). Then, live cells were selected based on negativity for the fixable viability dye, followed by gating for CD4^+^CD8^-^ T cells. CD4^+^ T cells were further subdivided into T regulatory cells (CD25^++^CD127^low/-^) and effector cells. Alternatively, CD4^+^ T cells were divided into naïve (CCR7^+^CD45RO^-^), central memory (CCR7^+^CD45RO^+^), effector memory (CCR7^-^CD45RO^+^) and terminally differentiated effector memory (CCR7^-^CD45RO^-^) subsets. The frequency of PD-1^+^ cells was determined for each of these subsets, and a representative example of PD-1 expression on total effector cells is shown in the figure.

**Figure S4**. *Gating strategy for T cell subsets based on cytokine expression.* (A) First, lymphocytes were selected based on their cell size (forward scatter; FSGA) and internal complexity or granularity (side scatter; SSGA). Then, single cells were selected based on proportional width (FSGW) and height (FSGH) and live T cells were selected based on negativity for the fixable viability dye and positivity for CD3, followed by gating for CD4^+^CD8^-^ T cells. (B) Cytokine expression by stimulated CD4^+^CD8^-^ T cells is shown for one representative control sample. (C) Unstimulated (biological control) samples (from the same donor) were used to set the gates for the stimulated samples.

**Figure S5**. *Gating strategy for cytokine expression by LPS-stimulated monocytes*. First, lymphocytes and monocytes were selected based on their cell size (forward scatter; FSC) and internal complexity or granularity (side scatter; SSC). Then, single cells were selected based on proportional area (FSC-A) and height (FSC-H) and live cells were selected based on negativity for the fixable viability dye. To exclude B cells, T cells, natural killer cells and neutrophils, cells expressing CD3, CD19, CD56, and/or CD66b were excluded. Monocytes were then selected based on positivity for HLA-DR and positivity for CD16 and/or CD14. Expression patterns of CD14/CD16, IL-1β, IL-6, and TNF-α are shown for a representative unstimulated (blue) and stimulated (red) control sample.

**Figure S6**. *Histograms for median fluorescence intensity levels of TLR2, TLR4 and CD86 expression by monocyte subsets*. For each surface marker, an overlay of histograms from classical (dark green), intermediate (light green), and non-classical (white) monocytes and T cells (grey; as negative control) from a representative control participant is shown.


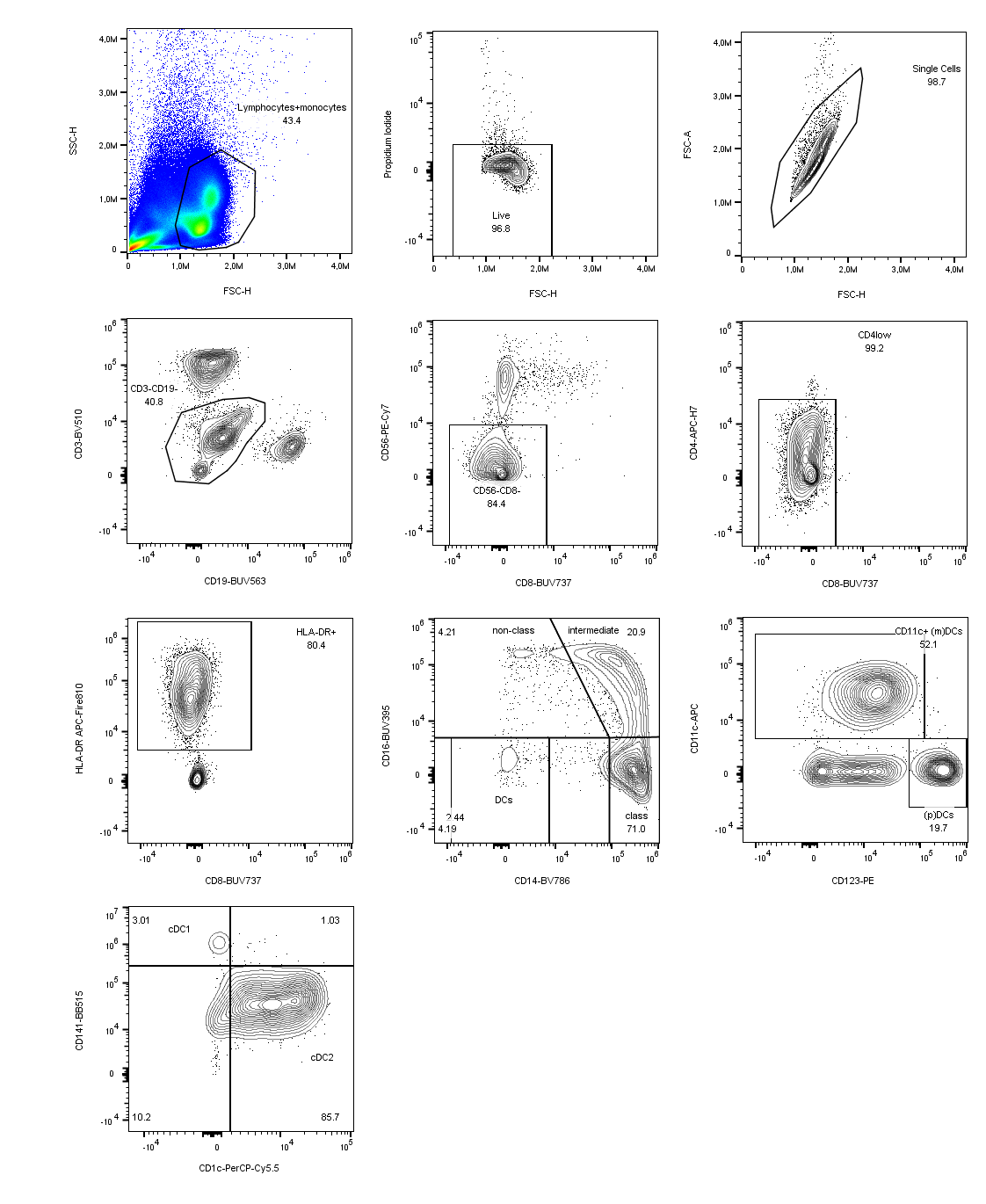


**Figure S1**. Gating strategy monocytes and dendritic cell subsets.


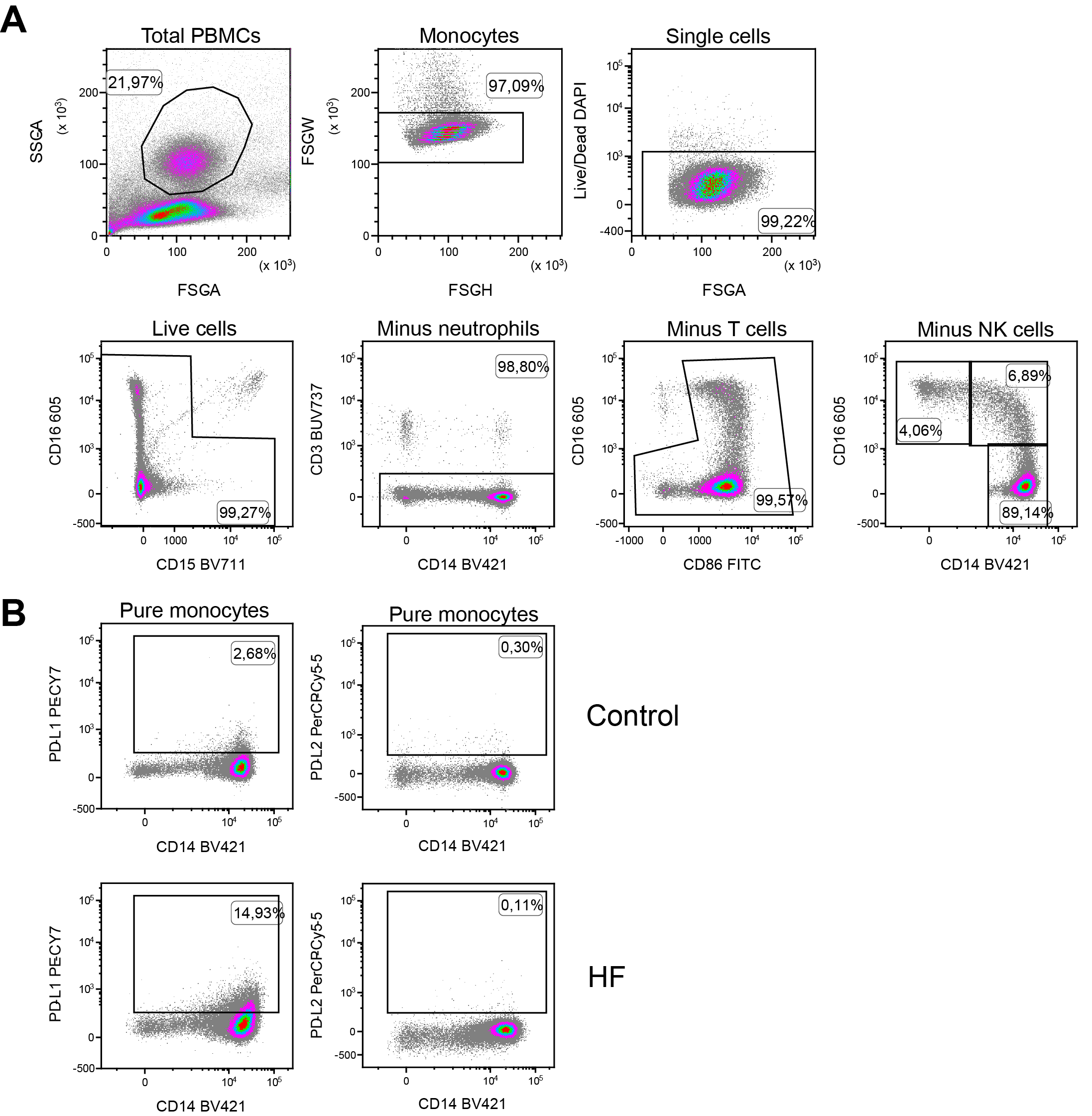


**Figure S2**. Gating strategy monocytes for assessment of PD-L1 and PD-L2 positive cells.


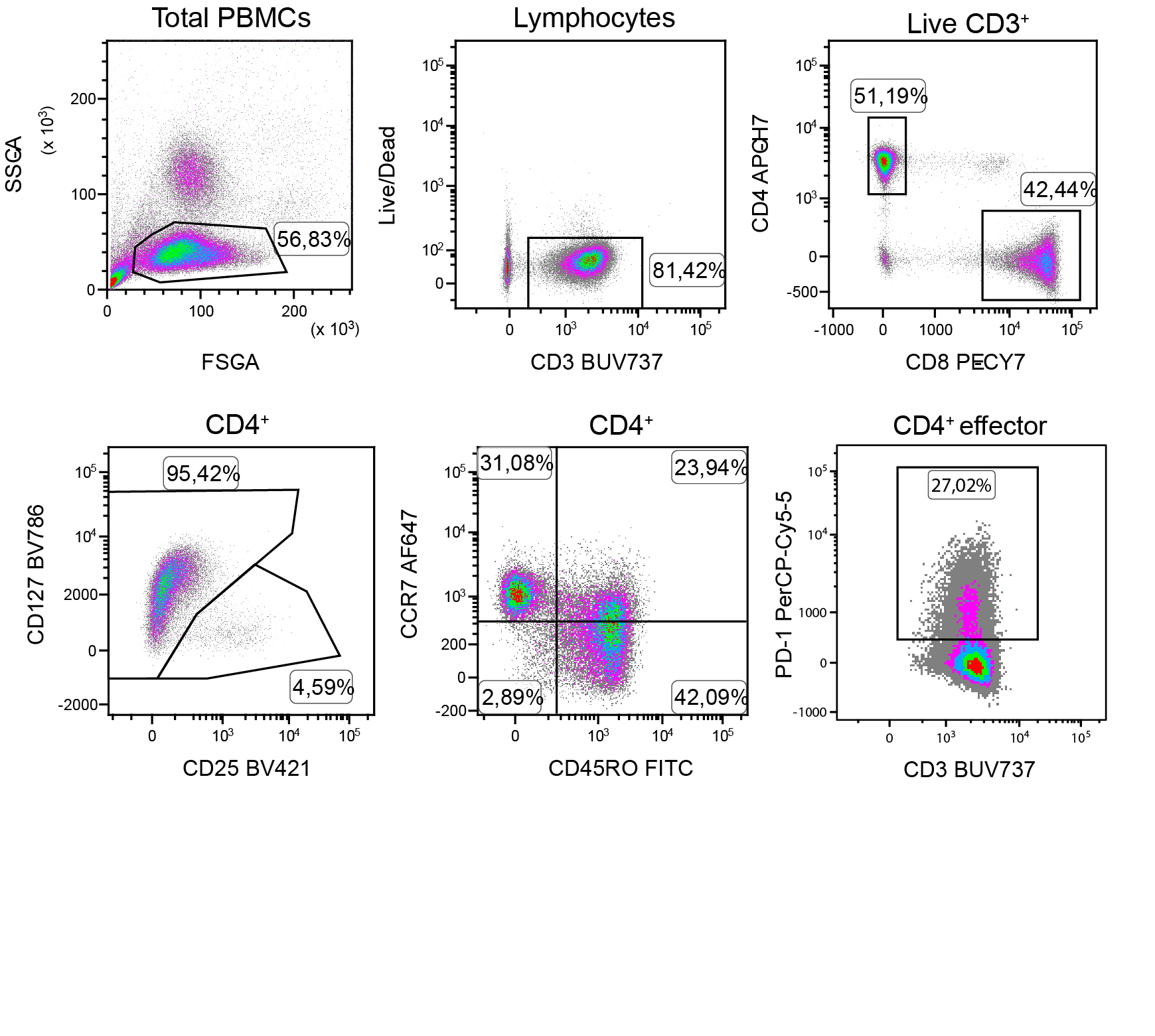


**Figure S3**. Gating strategy for T cell subsets based on surface receptor expression.


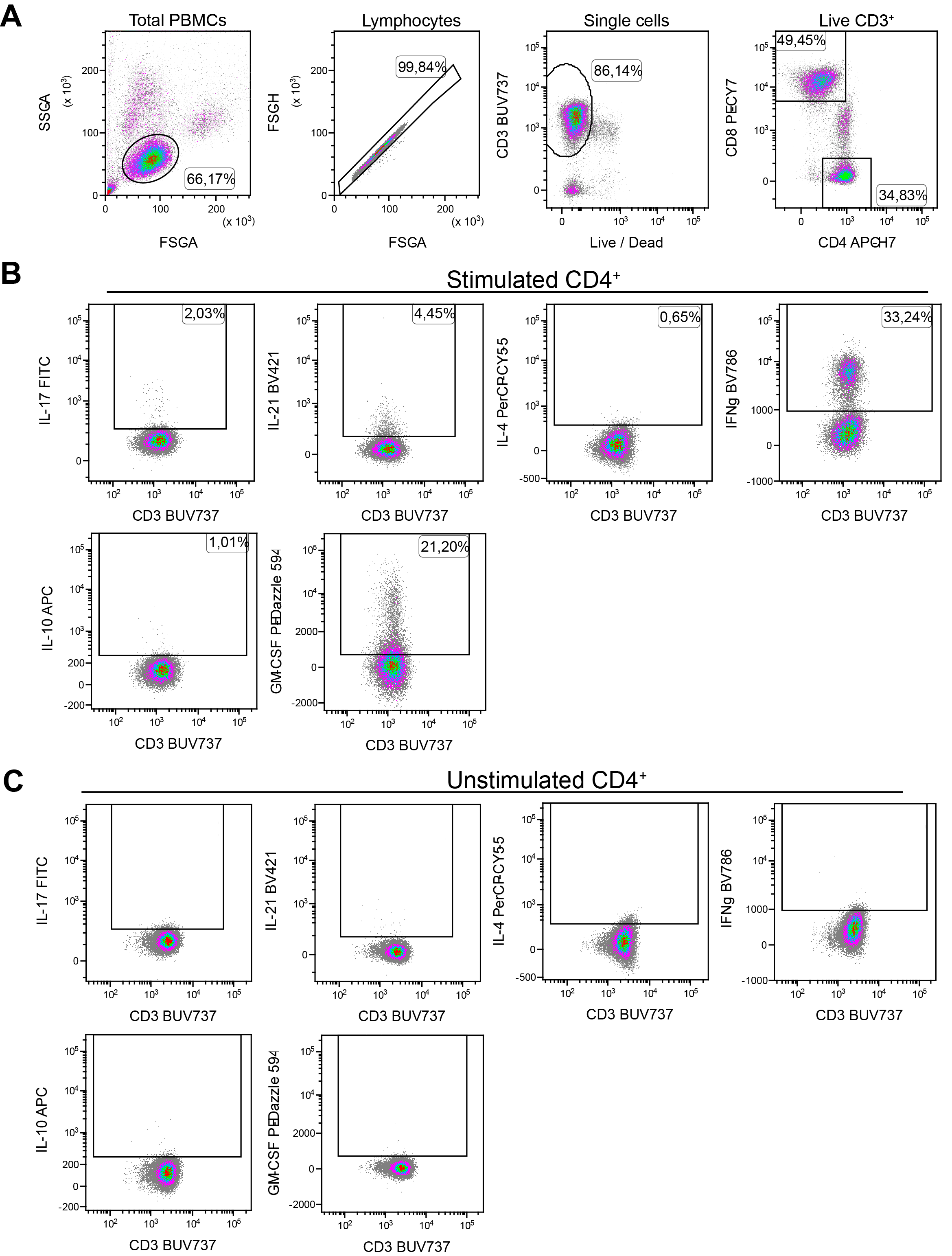


**Figure S4**. Gating strategy for T cell subsets based on cytokine expression.


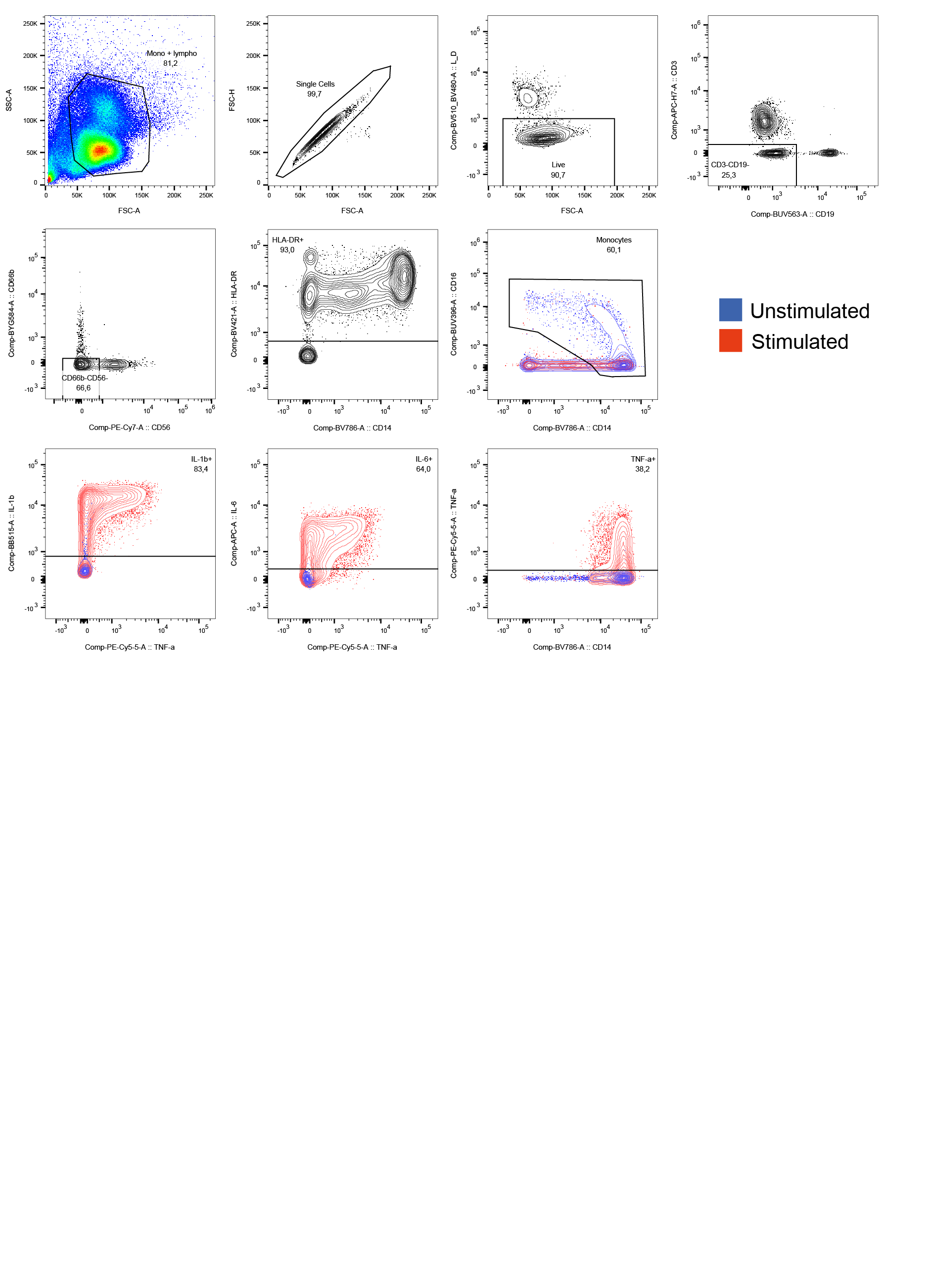


**Figure S5**. Gating strategy for cytokine expression by LPS-stimulated monocytes.


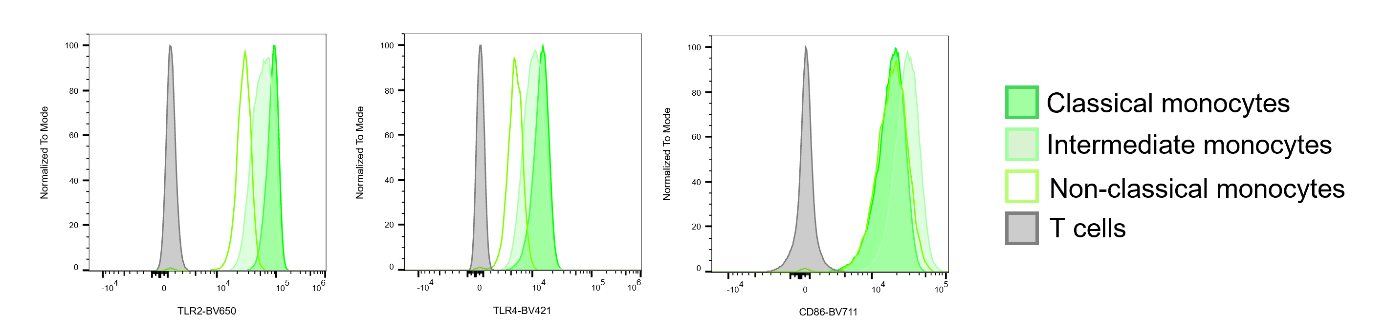


**Figure S6**. Histograms for median fluorescence intensity levels of TLR2, TLR4 and CD86 expression by monocyte subsets.
